# The Role of Mitochondria in Pyroptosis

**DOI:** 10.3389/fcell.2020.630771

**Published:** 2021-01-21

**Authors:** Qian Li, Nengxian Shi, Chen Cai, Mingming Zhang, Jing He, Ying Tan, Weijun Fu

**Affiliations:** ^1^Department of Emergency Medicine, Nanfang Hospital, Southern Medical University, Guangzhou, China; ^2^Department of Critical Care Medicine, Nanfang Hospital, Southern Medical University, Guangzhou, China; ^3^Department of Burns, Nanfang Hospital, Southern Medical University, Guangzhou, China

**Keywords:** mitochondria, MOMP, pyroptosis, gasdermin, apoptosis

## Abstract

Pyroptosis is a recently discovered aspartic aspart-specific cysteine protease (Caspase-1/4/5/11) dependent mode of gene-regulated cell death cell death, which is represented by the rupture of cell membrane perforations and the production of proinflammatory mediaters like interleukin-18(IL-18) and interleukin-1β (IL-1β). Mitochondria also play an important role in apoptotic cell death. When it comes to apoptosis of mitochondrion, mitochondrial outer membrane permeabilization (MOMP) is commonly known to cause cell death. As a downstream pathological process of apoptotic signaling, MOMP participates in the leakage of cytochrome-c from mitochondrion to the cytosol and subsequently activate caspase proteases. Hence, targeting MOMP for the sake of manipulating cell death presents potential therapeutic effects among various types of diseases, such as autoimmune disorders, neurodegenerative diseases, and cancer. In this review, we highlights the roles and significance of mitochondria in pyroptosis to provide unexplored strategies that target the mitochondria to regulate cell death for clinical benefits.

## Introduction

Mitochondria are the major sites of cellular energy production through oxidative phosphorylation (Jusic and Devaux, 2020). In addition to Adenosine Triphosphate (ATP) production, mitochondria are involved in various cellular processes, such as autoinflammatory response, cell differentiation, and immune regulation (West et al., 2011; Kasahara and Scorrano, 2014; Gurung et al., 2015; Weinberg et al., 2015). The effect of mitochondria in the types of cell death has attracted wide attention recently, but the mechanisms still seem obscure. Regulating cell death is a double-edged sword (Wang et al., 2020a). Excessive cell death will lead to many neurodegenerative diseases, such as Alzheimer disease and Parkinson disease. Inhibition of cell death is beneficial to the development of autoimmunity and cancer. Thereby, there's a lot of interest in targeting mitochondria to regulate cell death in diseases (Wang et al., 2020b). Apoptosis is a major type of cell death regulation, although the role of mitochondria on this type is not complete, but the effect of MOMP on apoptosis has got some progress (Tait and Green, 2010). MOMP occurs under the drive of some certain apoptosis-related protein molecules, such as BCL-2-associated X (BAX) and BCL-2 antagonist killer (BAK), which sequentially causes a series of cascades leading to cell death (Kale et al., 2018; Kalkavan and Green, 2018). However, other non-apoptotic signals can also cause MOMP, like pyroptosis signaling. Inflammasome mediated caspase-dependent cleaved fragment of gasdermin (GSDM) family can also be located to mitochondria to cause MOMP (Lee et al., 2019; Hu et al., 2020). In addition, this process also involves the opening of potassium efflux channels and the feedback to promote the formation of the inflammasome. It can be seen that mitochondria are involved in different types of cell death, although the specific roles and mechanisms are still poorly established.

Herein, we discuss the effect of mitochondria on pyroptosis, and highlight a new perspective on the interaction between mitochondrial apoptosis and pyroptosis. Combined with recent studies related to MOMP, we further discussed the interaction between MOMP in mitochondrial pyroptosis and apoptosis, and emphasized that targeting mitochondria may as a promising strategy to change the occurrence and development of diseases by regulating cell death.

## Types and Processes of Pyroptosis

Pyroptosis is a newly defined type of pro-inflammatory cell death in recent decades, which was originally considered as an inflammatory process before cell necrosis or apoptosis, but now it has been recognized as a cell death mode characterized by membrane perforation rupture and intracellular extravasation of inflammatory mediators (Zychlinsky et al., 1992; Fink and Cookson, 2005; Yuan et al., 2016). Currently, pyroptosis can be divided into three types according to different initiate activation modes, namely classical pyroptosis pathway, non-classical pyroptosis pathway, and apoptosis protein Caspase-3 mediated pyroptosis pathway (Kayagaki et al., 2015; Jorgensen et al., 2017; Wang et al., 2017). Although these three types have their own characteristics, they are related to each other. In addition, they share a common endpoint event which is to process IL-18 and IL-1β, activate the perforating protein GSDMD and eventually cause the cell membrane to break and release IL-18 and IL-1β (Ding et al., 2016; Kovacs and Miao, 2017).

## Regulation Mechanisms of Pyroptosis

The negative feedback regulation mechanism of pyroptosis itself will timely prevent the occurrence of it and inflammation (Frank and Vince, 2019). When caspase-1 is activated by different pathways, on the one hand, it continues to cleave its downstream signaling molecules including caspase-4/5/11, thus promoting the activation of GSDMD and the maturation and release of inflammatory factors. On the other hand, caspase-3/7 will also be non-specific activated when the pyroptosis occurs, and this kind of molecules will inactivate GSDMD by competitively cleaving it, playing a negative regulatory role to maintain the homeostasis (Takahama et al., 2018). Interestingly, when GSDMD was inactivated, cells switched from pyroptosis to apoptosis. In addition, TNF-αand some chemotherapy drugs can transform apoptosis to pyroptosis by cleaving GSDME. It can be seen that there is antagonism and conversion between pyroptosis and apoptosis through some unknown signaling pathways (Wang et al., 2017). In addition, some initial links of pyroptosis have the same trigger point as autophagy signaling pathway (Stocks et al., 2018). Many studies have shown that autophagy can negatively regulate pyroptosis (Schroder and Tschopp, 2010; Kim et al., 2015; Pu et al., 2017), and the mechanism may be that autophagy reduces the activation of inflammatory bodies by removing certain stimuli.

## Mechanisms of Mitochondrial Apoptosis

There are two kinds of apoptotic signals, death receptor pathway and mitochondrial pathway. The former occurs when the ligands outside the cell membrane bind to the receptors on the cell membrane, activating apoptosis executioner caspases (Caspase-3/7) through a series of cascade reactions, and finally leading to the activation of apoptosis (Boatright et al., 2003; Julien and Wells, 2017). The latter is derived from mitochondria. When cells are subjected to various pathological changes, such as the loss of certain growth factors and structural damage to genetic materials, the permeability of mitochondrial outer membrane increases and some soluble proteins in mitochondrial intermembrane space are released into the cytoplasm. Apoptotic signals will be then activated and cause cell death. As one of the main components of the electron transport chain, cytochrome-c is also a common soluble protein in mitochondria, which can be identified by apoptotic peptidase activating factor 1 (APAF1) to promote the formation of apoptotic bodies (Dorstyn et al., 2018). Subsequently, the initiator caspase 9 will be recognized and activated by the apoptosome. The next step is to cleave and activate apoptosis executioner caspases (Caspase-3/7), which is the common step between the two main apoptotic signaling pathways (Poreba et al., 2019). In addition, MOMP can induce cell apoptosis and death in a non-caspase-mediated way, which is related to the regulation of the B cell lymphoma 2 (BCL-2) protein family (Wei et al., 2001). The activation of BAK and BAX, some kinds of pro-apoptotic effectors, is essential for MOMP induced mitochondrial apoptosis (Lindsten et al., 2000; Ke et al., 2018). But only their specific interactions promote apoptosis, so BAK and BAX are also regarded as superfluous in some inappropriate conditions. For example, during the process of mitochondrial apoptosis, the mitochondrial membrane pore protein voltage-dependent anion-selective channel 2 (VDAC2) can associate with both two proteins, BAX is necessary for this process while BAK is not (Naghdi et al., 2015; Lauterwasser et al., 2016; Chin et al., 2018). Normally, BAK and BAX localize to the mitochondria and cytoplasm in an inactive form, respectively (Edlich et al., 2011; Schellenberg et al., 2013; Todt et al., 2015). During apoptosis, BAX moves toward the mitochondria and gets accumulation (Letai et al., 2002). Then BAK and BAX are activated by combining their hydrophobic bases with a subclass of BCL-2 homology regions (BH3)-only proteins (Leshchiner et al., 2013; Moldoveanu et al., 2013). After being activated, BAK and BAX can oligomerize each other, which is necessary for MOMP (Dewson et al., 2009, 2012; Bleicken et al., 2010; Subburaj et al., 2015).

There are some other effectors can also induce MOMP. For instance, BOK, a BAX/BAK-like BCL-2 protein, has been discovered can initiate MOMP and then commit cells to die without the regulation of BCL-2 proteins (Einsele-Scholz et al., 2016; Llambi et al., 2016; Fernández-Marrero et al., 2017). The proapoptotic characteristics of BOK could be explained by the instability of its own hydrophobic subunit (Zheng et al., 2018). In addition, some non-BCL-2 family proteins, such as GSDMD and GSDME, can also promote MOMP. Cleavaged by specific caspase, the amino-terminal of GSDMD and GSDME can not only locate to the cell membrane to cause plasma membrane permeabilization but also to the mitochondria to induce MOMP (Rogers et al., 2017, 2019; Wang et al., 2017). However, this direct way of MOMP mediated by gasdermin protein family needs further study. Indeed, there are some other types of cell death that are closely related to mitochondria. Mitochondria is the main source of intracellular reactive oxygen species, which can activate some receptor protein kinases and further form necrosome causing necroptosis (Schenk and Fulda, 2015; Zhang et al., 2017). Furthermore, reactive oxygen species can cooperate with iron ions to promote the catalytic reaction of lipid peroxides leading to ferroptosis (Dixon et al., 2012; Wang et al., 2016). Cell necrosis and ferroptosis are different types of cell death from apoptosis, and although some of the mechanisms are still unknown, this is sufficient to demonstrate the important role of mitochondria in the regulation of cell death.

## Interactions Between Pyroptosis and Mitochondrial Apoptosis

Pyroptosis is a newly discovered pro-inflammatory model of cell death initiated by the different inflammation-associated caspases. The inflammasome complex is assembled and activated under the stimulation of intra- and extracellular pathological signals, leading to the activation of inflammatory caspases. On the one hand, the activated caspase cleaves the precursor of inflammatory factors (IL-1β and IL-18) to promote its maturation; on the other hand, it also activates and cleaves GSDMD, leading to cell membrane pore formation and finally to lysis, cell content release and pyroptosis (Kayagaki et al., 2015; Shi et al., 2015; Broz and Dixit, 2016). As discussed earlier, the amino-terminal cleavage fragment of GSDMD can locate the mitochondria to cause MOMP, promoting the activation of caspase-3 (Rogers et al., 2019). Interestingly, caspase-3 is a executioner caspase during the activation of apoptosis. Furthermore, mitochondrial apoptosis can induce NLRP3 inflammasome mediated caspase-1 activity (Tsuchiya et al., 2019), which depends on caspase-3 mediated potassium channel glycoprotein activity. Potassium efflux from the cell via the channel, while this process should assist the assemblage of inflammasome. In addition, when GSDMD expression was low, the activation of caspase-1 tended to apoptosis rather than pyroptosis.

Another study has recently reported that another member of the gasdermin proteins family, GSDME, has the same function as GSDMD, and can also activate the intrinsic pathway downstream of inflammasome activation (Rogers et al., 2019). Briefly, GSDME is activated by caspase-3 to further generate the GSDME-N fragments. On the one hand, it can cause the pore-forming effect of cell membrane to mediate pyroptosis; On the other hand, it has been proved that GSDME-N can also cause changes in mitochondrial membrane permeability, further leading to the translocation of cytochrome-c from mitochondria to cytoplasm. While cytochrome-c can continue to activate apoptotic bodies and induce apoptosis, and the interaction between pyroptosis and apoptosis is just like a feedback regulation. Further researches should focus on the part of mitochondria to interfere with this feedback and thus influence the development of diseases associated with cell death patterns. Additionally, many studies in recent years have shown a complex link between mitophagy and pyroptosis. The current prevailing view is that there is a negative feedback regulation between mitophagy and pyroptosis (Yu et al., 2019; Davidson et al., 2020; Ding et al., 2020). Activation of caspase-1 caused by inflammasome would inhibit mitophagy and further enhance mitochondrial damage. In contrast, deletion of Parkin, a key regulator of mitophagy, would increase mitochondrial damage and promote pyroptosis (Yu et al., 2014). The mechanism may be related to the release of mitochondrial ROS and the disruption of membrane integrity mediated by pyroptosis. Moreover, potassium efflux and cytochrome-c also play important roles in the regulation of mitophagy and pyroptosis, but more details remain to be clarified. It can be seen that there are many crosstalks between mitochondrial apoptosis and pyroptosis, and a certain type of cell death cannot be emphasized alone, not just for mitochondrial apoptosis and pyroptosis.

## Conclusions and Perspectives

We have introduced the types and regulation mechanisms of pyroptosis briefly and discussed the significant effect of mitochondria on apoptosis in this review. In addition to the discussion of the mechanism between the well-known cell death type apoptosis and mitochondria, the MOMP-mediated apoptotic cell death in different signaling pathways was also be emphasized. According to recent findings, the association between MOMP and inflammasome-mediated pyroptosis was further highlighted, and the interplay between pyroptosis and apoptosis was also revealed. Although mitochondria are involved in a variety of regulatory cell death types, the molecular mechanisms involved are not completely exacted. Moreover, there are actually therapeutic drugs or molecules that target the mitochondria to regulate the pathological processes that involved mitochondria. Previous studie have ever reported that the permeability transition pore complex (PTPC), a multi-protein complex, is participated in the metabolism of mitochondrial stability and also in mitochondria-related intrinsic apoptotic pathways (Deniaud et al., 2006). This targeted intervention, which integrates multiple death signals, may be a promising therapeutic strategy for clinical application. Survivin, a member of the IAP5 gene family, has also been shown to act as a regulatory factor for mitochondrial apoptosis and to inhibit mitochondrial apoptosis by using adenovirus transduction technology in both animal and cell studies (Blanc-Brude et al., 2003). In addition, one homology domain of BCL-2 homology regions (BH3) Peptidomimetics can inhibit apoptosis and thus intervene in the progression of certain related diseases, although the development of targeted interventions is still limited (Nemec and Khaled, 2008). In summary, the targeted regulation of mitochondria and their related pathological processes has gradually aroused great interest. While further research and exploration are needed, this does not prevent the targeting of mitochondria as a new promising strategy to regulate cell death to achieve disease control or treatment of purposes.

## Author Contributions

QL, NS, and CC contributed to writing the manuscript. MZ and JH revised the manuscript. YT and WF revised the manuscript and contributed to concept of the manuscript. All authors approved this submission.

## Conflict of Interest

The authors declare that the research was conducted in the absence of any commercial or financial relationships that could be construed as a potential conflict of interest.
