# Expression of concern: The role of mitochondria in pyroptosis

**DOI:** 10.3389/fcell.2022.1014334

**Published:** 2022-08-17

**Authors:** Frontiers Editorial Office

Following publication, the publisher has uncovered conclusive evidence that a false identity was used as a peer reviewer for this article. This reviewer was not suggested by the authors. This peer reviewer has now been removed.

This article is currently under post publication assessment. This expression of concern has been posted while Frontiers awaits the outcome of this assessment and will then be updated accordingly.

